# Power-law correlations and coupling of active and quiet states underlie a class of complex systems with self-organization at criticality

**DOI:** 10.1051/epjconf/202023000005

**Published:** 2020-03-11

**Authors:** Fabrizio Lombardi, Jilin W.J.L. Wang, Xiyun Zhang, Plamen Ch Ivanov

**Affiliations:** 1 Institute of Science and Technology Austria, A-3400 Klosterneuburg, Austria; 2 Keck Laboratory for Network Physiology, Department of Physics, Boston University, Boston, MA 02215, USA; 3 Harvard Medical School and Division of Sleep Medicine, Brigham and Women Hospital, Boston, MA 02115, USA

## Abstract

Physical and biological systems often exhibit intermittent dynamics with bursts or avalanches (active states) characterized by power-law size and duration distributions. These emergent features are typical of systems at the critical point of continuous phase transitions, and have led to the hypothesis that such systems may self-organize at criticality, i.e. without any fine tuning of parameters. Since the introduction of the Bak-Tang-Wiesenfeld (BTW) model, the paradigm of self-organized criticality (SOC) has been very fruitful for the analysis of emergent collective behaviors in a number of systems, including the brain. Although considerable effort has been devoted in identifying and modeling scaling features of burst and avalanche statistics, dynamical aspects related to the temporal organization of bursts remain often poorly understood or controversial. Of crucial importance to understand the mechanisms responsible for emergent behaviors is the relationship between active and quiet periods, and the nature of the correlations. Here we investigate the dynamics of active (*θ*-bursts) and quiet states (*δ*-bursts) in brain activity during the sleep-wake cycle. We show the duality of power-law (*θ*, active phase) and exponential-like (*δ*, quiescent phase) duration distributions, typical of SOC, jointly emerge with power-law temporal correlations and anti-correlated coupling between active and quiet states. Importantly, we demonstrate that such temporal organization shares important similarities with earthquake dynamics, and propose that specific power-law correlations and coupling between active and quiet states are distinctive characteristics of a class of systems with self-organization at criticality.

## 1 Introduction

Bursting dynamics is ubiquitous across systems operating far from equilibrium. From earthquakes to neuronal and physiologic networks, the dynamics of such systems can be described as the irregular alternation of active and quiet states. Depending on the particular systems, active states are characterized as bursts [1], avalanches [2], flares or earthquakes [3], and exhibit power-law size and duration distributions. The emergence of such characteristics, which are typical of systems at the critical point of a second order phase transition, is considered a fingerprint of self-organization at criticality [4]. The concept of self-organized criticality (SOC) was introduced by Bak, Tang and Wiesenfeld to explain emergent power-law behaviors in far-from-equilibrium systems. They proposed that such systems may self-organize at criticality through slow accumulation and fast redistribution of energy, as exemplified by the sandpile model [5].

The framework of SOC has been used to characterize many physical and biological systems [6]. Recent empirical results suggest that the brain may also operate at criticality [2, 7–10]. While static properties as the power-laws of the size and duration distributions have been widely investigated in this context [2, 11], dynamical aspects and mechanisms leading to the emergent critical behavior remain poorly understood. Here we study the temporal organization of cortical activity across the sleep-wake cycle of rats, with a particular focus on the correlation and coupling underlying the emergent critical dynamics of active and quiet states.

The sleep-wake cycle of rats is largely dominated by the *δ* and *θ* rhythms. During NREM sleep, cortical activity is characterized by *δ* rhythm, low-frequency high-amplitude oscillations referred to as slow-wave activity [12], whereas REM sleep and arousals/wake state are characterized by *θ* rhythm, desychronized and localized oscillations of higher frequency and lower amplitude [13]. Thus, *θ*-bursts can be interpreted as active states and *δ*-bursts as quiet states of the brain activity in the sleep-wake cycle. This interpretation is consistent with the basic neurophysiological understanding of *δ* rhythm as the cortical default mode [14–16]. In contrast, oscillations in the *θ* band are associated with activated state, such as REM, arousals and wakefulness [17, 18].

We analyze long-term continuous EEG recordings in rats, and dissect emergent signatures of criticality in the dynamics of *θ*- and *δ*-bursts in relation to their correlation properties and reciprocal coupling. We show that *θ*-burst (active states) durations follow a power-law distribution while the δ-burst (quiet states) durations follow an exponential-like behavior [1]. Importantly, we demonstrate that both active and quiet state durations are long-range power-law correlated, and that the observed temporal organization implies the existence of an anti-correlated coupling between active and quiet states. Finally, the analysis we present uncovers a striking parallel with earth-quakes dynamics, suggesting that specific power-law correlations and coupling between active and quiet states are distinctive characteristics of a class of systems with self-organization at criticality.

## 2 Results

### 2.1 Critical dynamics of active and quiet states across the sleep-wake cycle

Cortical EEG signals were recorded continuously for 48 h (2 days, 12 h dark and 12 h light) in 10 rats. The reader may refer to [1] for further details on the experimental design and data collection.

**Figure 1.**
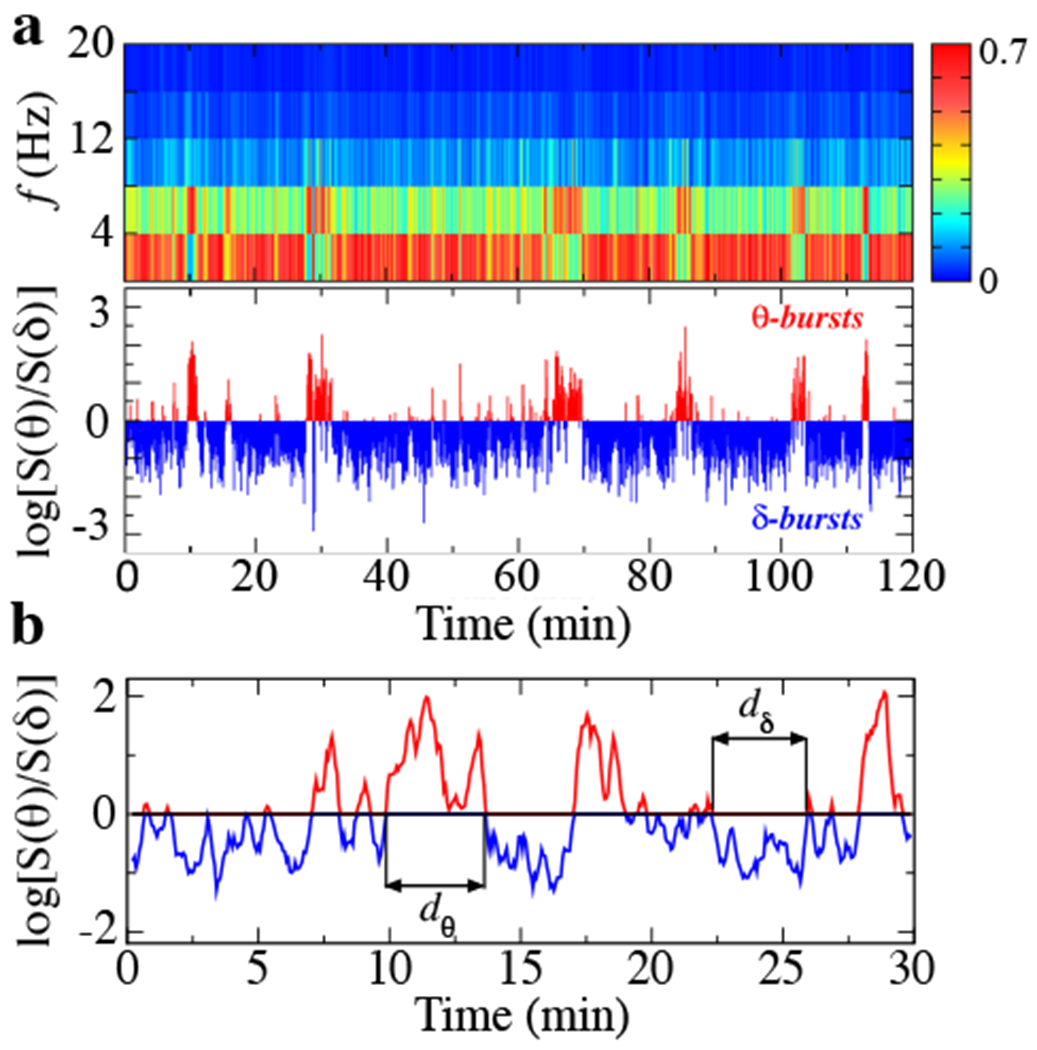
Intermittent transitions between distinct dominant cortical rhythms across the sleep-wake cycle. (a) (Top panel) Spectrogram derived from a 2 h segment of the EEG signal recorded from a rat during a 12 h light period. Spectral power is calculated in non-overlapping time windows *w* = 5 s for physiologically relevant frequency bands between 0.5 Hz and 20 Hz. Segments in red indicate bursts of prominent activity in the low frequency band corresponding to *δ* rhythm (0-4 Hz), and in the frequency band corresponding to *δ* rhythm (4 - 8 Hz). (Bottom panel) Ratio *R*_*θδ*_ = *S* (*θ*)/*S*(*δ*) of the spectral power in the *θ* and *δ* band in log-arithmic scale obtained from the spectrogram in top panel. Values *R_θδ_* above *log(Th)* = 0 (*Th* = 1) indicate periods with dominant *θ* rhythm (in red), while values below *log(Th)* = 0 correspond to predominance of *δ* rhythm (in blue). (b) Smoothed ratio *R_θδ_* of the spectral power in the *θ* and *δ* band during 30 min segment of 12 h dark (lights-off) period. *θ*- and *δ*- bursts are defined as sequences of consecutive windows where either the power in *θ* or *δ* band is dominant, and are labeled as *d_θ_* and *d_δ_*. *R_θδ_* is calculated on non-overlapping windows *w* = 5 s and the smoothing is performed using a 5 point moving average.

To characterize the micro-dynamics of dominant brain rhythms across the sleep-wake cycle, we divide the EEG signal in *N* non-overlapping windows of length *w* and evaluate the spectral power in each window for several frequency bands comprised between 0.5 and 20 Hz (Fig. 1). In Fig. 1a we show a typical spectrogram *S_f_(t)* as a function of time for a 2 h EEG recording. The spectral power *S_f_(t)* is mostly concentrated in the low frequency bands *δ* (0 - 4 Hz) and *θ* (4 - 8 Hz), and exhibits sharp transitions from periods with dominant *δ* to periods with dominant *θ* waves. We then consider the ratio *R_θδ_* = *S(θ)/S(δ)* between *θ* and *δ* power (Fig. 1a), whose intennittent fluctuations between values larger and smaller than a threshold *Th* = 1 captures the alternation between periods with dominant *θ*-waves, *R_θδ_* > *Th* = 1, and periods with dominant *δ*-waves, *R_θδ_* < *Th* = 1. We define bursts in *θ* and *δ* rhythms as sequences of consecutive time windows where *R_θδ_* > *Th* = 1 and *R_θδ_* < *Th* = 1, respectively (Fig. 1b). The duration *d* of a burst is defined as *d* = *n* * *w*, where *n* is the number of consecutive windows belonging to a given burst and *w* is the window length (Fig. 1b).

**Figure 2.**
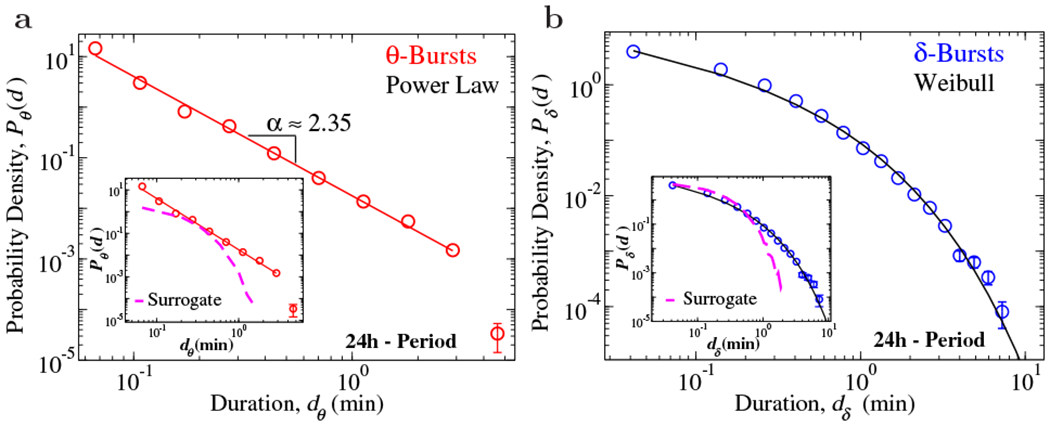
Durations of active (*θ*-bursts) and quiet states (*δ*-bursts) across the 24 h sleep-wake cycle follow distinct probability distributions indicative of self-organization at criticality. (a) Distribution of *θ*-burst durations over the 24 h period (pooled data, 10 rats) exhibits a power-law behavior (colored tick lines) with *α* = 2.34 ± 0.06. (b) Distribution of dburst durations for control over 24 h period (pooled data, 10 rats) follow a Weibull distribution with *β* = 0.59, *λ* = 0.16. The black tick line is a Weibull fit of the distribution. Durations are calculated using a window size *w* = 5 s [1], and threshold *Th* = 1 on the ratio *R_θδ_* (Fig. 1). Insets: Distributions of surrogate *θ*- and *δ*-burst durations markedly deviates from the original distributions. Error bars *δP* are calculated for each value of the distributions as $\delta P=(\sqrt{p(1-p)/N})/dD,$ and where not shown are smaller than the symbol size. Error bars calculation and binning procedure are described in [1].

Next, we study the probability distributions *θ* and *δ* burst durations (Fig. 2). We find that the distribution *P_θ_* of *θ*-burst durations recorded in a 24 h period exhibits power-law behavior (Fig. 2a),

(1) $$P_{\theta }(d)\;\alpha \;d^{-\alpha }.$$

In contrast, the distribution *P*_*δ*_ of *δ*-burst duration is described by a Weibull distribution

(2) $$P_{\delta }(d;\lambda ,\beta )=\frac{\beta }{\lambda }{\left(\frac{d}{\lambda }\right)}^{\beta -1}e^{-{\left(d/\lambda \right)}^{\beta }},$$

where *λ* indicates the characteristic time scale, and *β* is the shape parameter (Fig. 2c, d). Surrogate tests perfonned by randomizing the sequence of windows *w* in the EEG spectrogram (Fig. 1a) leads to exponentially distributed *θ*-and *δ*-burst (Fig. 2, insets), and indicate that the observed temporal organization in bursting activity of brain rhythms is physiologically relevant and relates to underlying regulation.

This coexistence of scale-free *θ*-burst and exponential-like distributed *δ*-burst durations shares striking similarities with non-equilibrium phenomena exhibiting self-organized criticality [5]. In that context, bursts constitute the active phase of the process and follow power-law statistics [4, 6]. Consecutive bursts are separated by inactive phases or quiescent periods whose distribution depends on the details of the system and generally exhibit an exponential tail [19–22], and is an exponential for the paradigmatic sandpile model of self-organized criticality [23].

The duality of power-law and Weibull distribution in the bursting dynamics of *θ* and *δ* rhythms is closely reminiscent of this scenario, where scale-free *θ*-bursts in cortical activity can be seen as avalanches or earthquakes (active states), while *δ*-bursts can be interpreted as the quiet periods between active states. This interpretation is consistent with the basic neurophysiological understanding of *δ* rhythm as the cortical default mode [14–16], and *θ* rhythm as oscillations associated with activated state, such as REM, arousals and wakefulness [17, 18]. Due to the respective amount of wakefulness and REM sleep in our data, most of the analyzed *θ*-bursts are likely associated with arousals and wake [1].

Given this analogy, in what follows we will refer to *θ*-bursts as active states, and *δ*-bursts as quiet states.

### 2.2 Scale-invariant critical behavior of active and quiet states across time scales

We have shown that active and quiet states exhibit distinct duration distributions: A power-law for the active states, indicating absence of a characteristic time scale, and a Weibull for *δ*-bursts, with a characteristic time scale λ. In our burst analysis we introduced two parameters, the window size *w* and the threshold *Th* that were (Fig. 1). In the previous Section we presented results based on a particular observational window size *w* and threshold *Th*. To demonstrate such results are independent of the particular choice of *Th* and *w*, we repeat the analyses for a range of parameter values. We find that the dynamics of burst durations across the 24 h sleep-wake cycle is indeed described by unique scaling functions.

**Figure 3.**
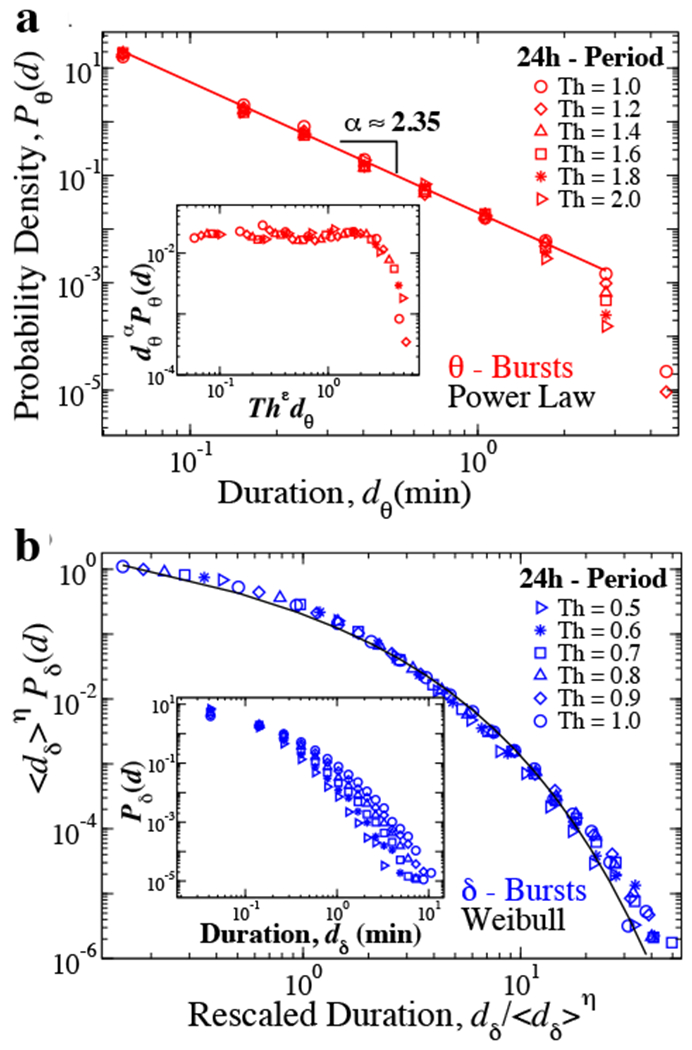
Duration distributions of active and quiet states are independent of threshold *Th* utilized to define bursts and are described by unique scaling functions. (a) Probability distributions of *θ*-burst durations over a 24 h period (pooled data, 10 rats) evaluated using different *Th* values consistently follow the same power law behavior (red line) reported in Fig. 2, with a cut-off that is controlled by *Th*. With increasing *Th* the distribution cut-off shifts towards shorter burst durations. Insets: Data for different *Th* collapse onto a single universal function *f_θ_* when we plot *P(d)d^α^* versus *Th*^*∊*^*d*, with *α* = 2.35 (a) and ∊ = 0.8. (b) Rescaled distribution of *δ*-burst durations for control rats over a 24 h period (pooled data) obtained for different *Th* values collapse onto a single function following a Weibull behavior *f(d; λ,β)* (black line), with *λ* = 0.55 and *β* = 0.59. Distributions are rescaled by ⟨*d*_δ_⟩^*η*^, where *⟨dδ⟩* is the mean *δ*-burst duration and *η* = 1.2. Inset: Distributions *P_δ_* for different thresholds (not rescaled). Results in all panels are obtained for a fixed scale of analysis, keeping the window size *w* = 5 s (Fig. 1). Results are consistent when considering separately light and dark periods [1].

We first examine the duration distributions of *θ*- and *δ*- bursts for different threshold values *Th*, keeping the window size *w* fixed. By increasing the threshold on the ratio *R_θδ_* from *Th* = 1 to *Th* = 2, we find that the scaling exponent *α* characterizing the power-law distribution of active state durations remains stable, as demonstrated by the data collapse in Fig. 3. The scaling behavior is followed by a cutoff that, with increasing *Th* values, shifts to shorter burst durations *d*_*θ*_. This behavior can be expressed in terms of the following scaling relation,

(3) $$P_{\theta }(d)=d^{-\alpha }f_{\theta }(d/Th^{-\in }).$$

where *f(d/Th^−∊^)* is a scaling function, and *∊* expresses the dependence of the cutoff on *Th*. The existence of a scaling function *f(d/Th^−∊^)* satisfying Eq. 3 is confirmed by the data collapse obtained by plotting *P(d)d^α^* versus *Th^∊^d* for several values of *Th* (inset in Fig. 3).

Similarly, we show that the distribution of quiet state durations is independent of the threshold *Th*, and is described by a single scaling function (Fig. 3). Since the quiet states correspond to periods with dominant *δ*-waves and are defined by a sequence of windows *w* where *R_θδ_* < *Th* = 1 (Fig. 1), to explore the behavior of the duration distribution for states with increasingly dominant *δ* power, we repeat the analysis for different values *Th* < 1. We observe that, as *Th* decreases, the distributions change: The probability for long quiet state decreases, while short quiet states become more likely (insets in Fig. 3). However, when distributions are rescaled by their respective mean quiet state duration *⟨d_δ_⟩*, they all collapse onto a unique function *f*_δ_. Such function is defined by the scaling relation

(4) $$P_{\delta }(d)={\langle d_{\delta }\rangle }^{-\eta }\cdot f_{\delta }(d/{\langle d_{\delta }\rangle }^{\eta })$$

where *η* = 1.2, and is well described by a Weibull functional form (Fig. 3).

**Figure 4.**
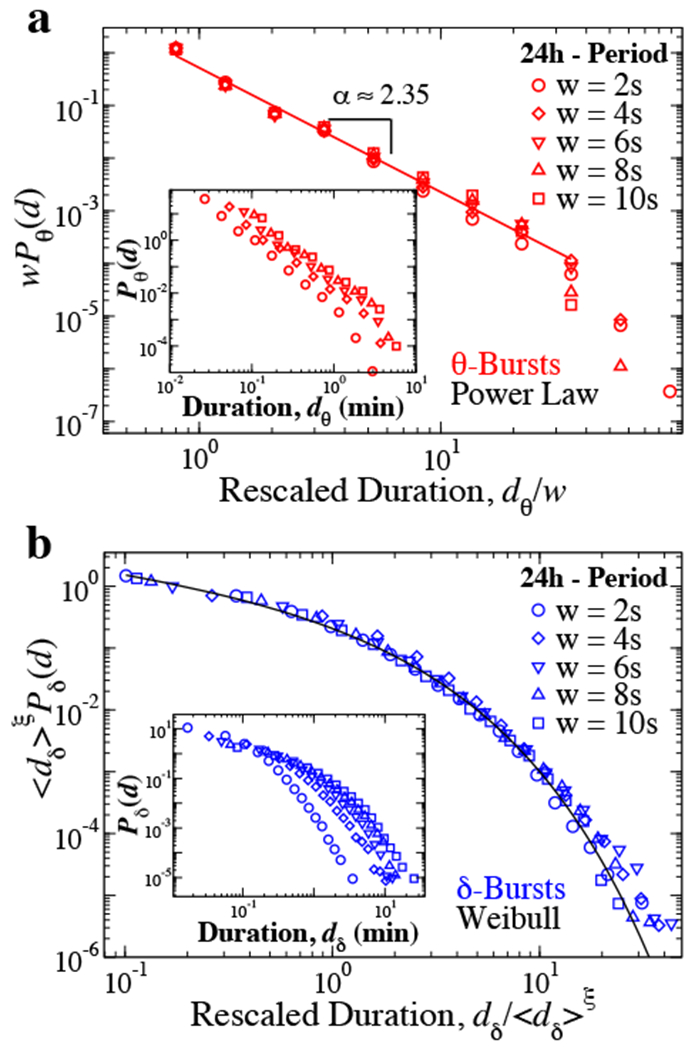
Active and quiet states duration distributions are independent of the scale of analysis defined by the window size *w*. (a) Probability distributions of *θ*-burst durations over a 24 h period (pooled data, 10 rats) evaluated using different values of the window size *w* follow the power law behavior (red line) reported in Fig. 2, as proven by the data collapse. Distributions are rescaled by the window size *w* and consistently show the same power law behavior (red line) with *α* ≈ 2.35. Insets: Distributions *P_θ_* for different window sizes *w* (not rescaled). (b) Rescaled distributions of *δ*-burst durations over a 24 h period (pooled data, 10 rats) obtained using different *w’*s collapse onto a single function following a Weibull behavior *f(d; λ,β)* (black line). Distributions are rescaled by ⟨*d*_δ_⟩^ξ^ where ⟨*d*_δ_⟩ is the mean *δ*-burst duration and *ξ* = 1.2. Inset: Distributions *P_δ_* for different thresholds (not rescaled). Results in all panels are obtained for fixed threshold *Th* = 1 on the ratio *R_θδ_* (Fig. 1). Results are consistent when considering separately light and dark periods [1].

Next, we investigate whether the functional behavior of the distributions depends on the window size *w*. Intuitively, larger *w*’s would tend to fail in identifying short bursts and merge them together, thus causing an increase in the probability of observing longer durations. In particular, larger *w*’s should influence the active state (*θ*-burst) power law behavior and lead to a decrease of the exponent *α*. Indeed, the window size *w* mainly influences the tail of the distributions, as shown in Fig. 4. When one rescales the durations by the window size *w*, the distributions collapse onto a single curve with small deviation on the tail due to the large window effect for *P_θ_*(*d; w* > 6) (Fig. 4).

Such rescaling is defined by the following relation

(5) $$P_{\theta }(d)∼w^{-1}\cdot f_{\theta }(d/w).$$

A similar data collapse characterizes the dependence of the quiet state (*δ*-burst) duration distribution on window size *w*. We observe that for increasing *w* the probability for long *δ*-bursts increases, while short *δ*-bursts become less likely (insets in Fig. 4). When quiet state duration distributions corresponding to different window sizes *w* are rescaled by their respective mean duration ⟨*d_δ_*δ, we find that all distributions collapse onto a unique function *f_δ_* following a Weibull behavior (Fig. 7b,d) and obeying the scaling relation

(6) $$P_{\delta }(d)∼{\langle d_{\delta }\rangle }^{-\xi }\cdot f_{\delta }(d/{\langle d_{\delta }\rangle }^{\xi }).$$

where ξ = 1.2 (Fig. 4).

Repeating the analysis or 12-hour dark and light periods separately, we find that Eq. 3, Eq. 4, 5 ,and Eq. 6 consistently describe the dynamics of *δ*- and *θ*-bursts[1].

### 2.3 Earthquake-like architecture in the temporal organization of active and quiet states

**Figure 5.**
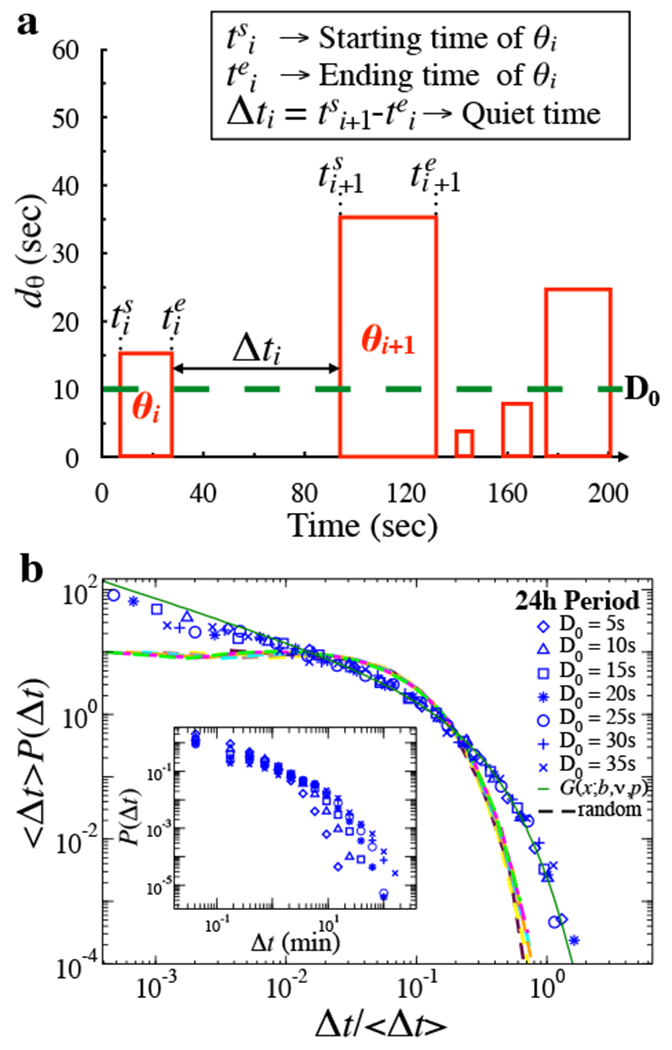
Earthquake-like temporal architecture of quiet times between consecutive *θ*-bursts across observational scales. (a) Schematic diagram of quiet times ∆*t* between consecutive *θ*-bursts. A quiet time ∆*t_i_* is the time elapsed from the end of burst *θ*_*i*_ to the beginning of the following burst *θ*_*i*+1_. (b) Distribution of quiet times for different thresholds *D*_0_ on *θ*-burst durations over a 24 h period collapse onto a unique function when rescaled by the average quiet time ⟨∆*t*⟩ (main panel). The scaling function is well described by a generalized Gamma distribution *G(x; b, v, p)* (solid green line), with the following set of parameters: *b* = 2.03, *v* = 0.30, *p* = 0.81. Applying the same procedure to a sequence of randomly reshuffled *θ*-burst durations leads to distributions that collapse onto an exponential function (dashed lines). Insets: Distributions of quiet times for different thresholds *D*_0_ before rescaling. Insets: Distributions of quiet times for different thresholds *D*_0_ before rescaling. Results are consistent when considering separately light and dark periods [1].

**Figure 6.**
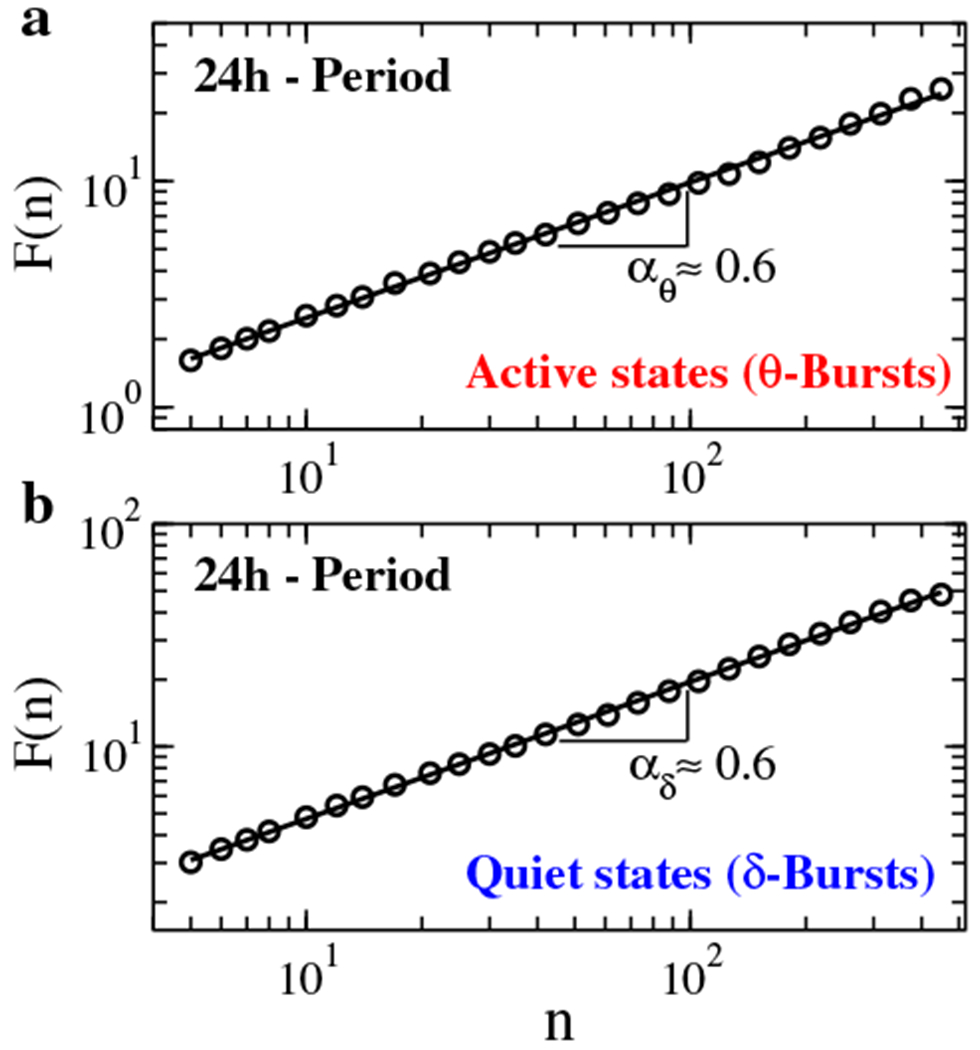
Long-range power-law correlations in sequences of consecutive *θ*- and *δ*-burst durations are key elements of self-organization at criticality in brain activity. Detrended fluctuation analysis for sequences of *θ*-bursts (a) and *δ*-burst (b) durations. Burst durations are calculated using a window *w* = 5 s and threshold Th = 1 on the ratio *R_θδ_* (Fig. 1). The root mean square (r.m.s.) fluctuation function *F(n)* is obtained averaging over all rats. Log-log plots of *F(n)* vs the time scale of analysis *n*, where *n* is the number of consecutive burst durations, show power-law relations *F(n) α n^αd^* over a broad range of scales *n*. The scaling exponents are significantly larger than 0.5 (α_*θ*_ = 0.599 ± 0.004 and *α*_*δ*_ = 0.615±0.003), indicating presence of positive (persistent) long-range correlations in both active and quiet states.

To further characterize the temporal organization of active and quiet states, we investigate the relationship between the duration of *θ*-bursts and their temporal occurrence (Fig. 5). To this end, we consider the sequence of *θ*-bursts and we study the statistical features of the quiet times ∆*t* separating consecutive bursts as a function of the scale of analysis, which is controlled by a threshold *D*_0_ on the *θ*-burst durations. This procedure corresponds to the analysis of earthquake catalogs at different magnitude thresholds [20, 21]. We define the quiet time ∆*t_i_* as the period from the end of *θ*_*i*_-burst to the beginning *θ*_*i*+1_-burst. Thus, the statistical characteristics of ∆*t_i_*, depend on the threshold value *D*_0_. We then obtain the probability distribution *P*(∆*t; D*_0_) of quiet times ∆*t_i_* for different values of *D*_0_. For *D*_0_ = 0, the quiet times correspond to the previously analyzed *δ*-bursts or quiet states. With increasing threshold (scale of observation) *D*_0_, the probability of longer ∆*t_i_* increases, while the probability of short ∆*t_i_* decreases, leading to different curves for the distributions *P*(∆*t; D*_0_) (insets of Fig. 5). Remarkably, by rescaling each distribution by the corresponding average quiet time ⟨∆*t*⟩_*D*_0__, we find that all curves collapse onto a single function *G* (Fig. 6c, d), defined by the following scaling relation

(7) $$P(\Delta t)={\langle \Delta t\rangle }^{-1}\cdot G(\Delta t/\langle \Delta t\rangle ),$$

with the scaling function *G*(∆*t*/⟨∆*t*⟩) well described by the generalized Gamma distribution *G*(∆*t*/⟨∆*t*⟩; *b, v, p*) = *p/b*^*v*^(∆*t*/⟨∆*t*⟩)^*v*−1^ *e*^−(∆*t/b*⟨∆*t*⟩*t*)^*P*^^ /Γ(*v/p*) [1].

This evidence draws a strong parallel with the dynamics of earthquakes. Indeed, time intervals between consecutive earthquakes also follow a generalized Gamma distribution, independently of the geographical locations and minimum magnitude thresholds [3, 21]. Importantly, the presence of a non-exponential scaling function for the quiet times indicates specific temporal order in the occurrence of *θ*-bursts, which is independent of the scale of observation. To explicitly verify this, we randomly reshuffle the sequence of *θ*-burst durations, while preserving the sequence of *δ*-bursts (quiet states) durations corresponding to quiet times at *D*_0_ = 0, and we perfonn the analysis on the reshuffled sequence to obtain quiet time distributions *P_rand_*(∆*t; D*_0_) for different thresholds *D*_0_. In this case, after rescaling the distributions *P*_*rand*_(∆*t; D*_0_) by the average quiet time ⟨∆*t*⟩_*D*_0__, their curves collapse onto an exponential distribution (dashed lines in Fig. 5), indicating temporal independence between consecutive events [24]. This clearly demonstrates that temporal correlations are intimately related to the existence of non-exponential scaling functions (Eq. 7) [21, 24], and indicates the presence of a certain temporal order in *θ*-bursts occurrence and coupling between *θ*-bursts and quiet times [25].

### 2.4 Long-range power-law correlations in the durations of *θ* and *δ* bursts

Thus, we first perfonn conelation analysis to quantify long-range features in the temporal organization of *δ*- and *θ*-burst durations. To this end, we utilize the detrended fluctuation analysis (DFA), a method specially tailored to quantify long-range power-law conelations embedded in non-stationary signals with bursting dynamics and polynomial trends [26–29]. The DFA is based on evaluation of the root mean square (r.m.s.) fluctuation function *F(n)*, where *n* is the scale of analysis expressed in number of consecutive bursts (Fig. 6). A scaling relationship of the form *F(n) α n^αd^* indicates presence of long-range power-law correlations in the time series of burst durations if *α_d_* Φ 0.5. An exponent *α_d_* ∊ [0, 0.5) indicates anti-correlations (where short burst durations tend to be followed by longer burst durations), while *α_d_* ∊ (0.5,1] indicates positive persistent correlations (long bursts tend to be followed by longer bursts); *α_d_* = 0.5 corresponds to a random walk and absence of correlations.

We perfonn DFA on sequences of *θ*- and *δ*-burst durations separately, and find that both *θ*- and *δ*-bursts exhibit long-range power-law correlations with an exponent *α_d_* ≃ 0.6 (Fig. 6). Similar exponents characterize the correlations during dark and light periods [1], indicating a basic property of burst correlations, independent of the dominant physiologic state (i.e. sleep or wake).

### 2.5 Anti-correlated coupling between the durations of consecutive *θ* and *δ*-bursts

**Figure 7.**
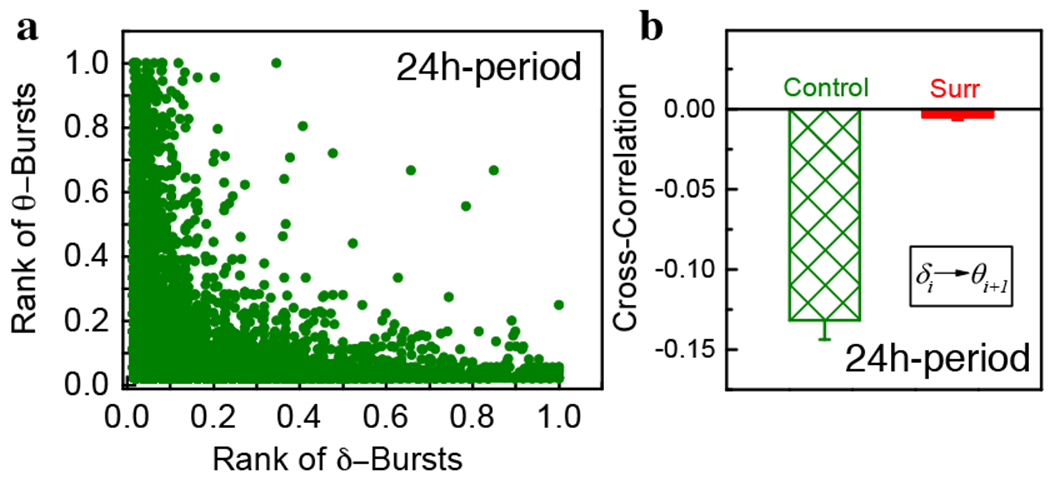
Anti-correlated coupling between *<*5- and *θ*-burst durations is an essential dynamical feature of brain criticality. Scatter plots and rank correlation analysis demonstrate anti-correlated coupling between consecutive *δ*- and *θ*-burst durations. (a) Scatter plot of *θ*-burst ranks vs *θ*-burst ranks. Each dot represents a pair formed by a *δ*-burst and the following *θ*-burst. (b) Average Spearman’s cross-correlation coefficient in 24h period (10 rats) (green bar). The correlation coefficients is significantly different from the corresponding values obtained in the surrogates (red bars) after randomly reshuffling the original order of *θ*- and *δ*-bursts (*t-test: p* < 0.001). All durations are calculated using a window *w* = 5 s and threshold *Th* = 1 on the ratio *R_θδ_* (as in Fig. 1).

Next, we investigate the coupling between consecutive *δ*-and *θ*-burst durations. We focus on the relationship between ranks of consecutive *δ*- and *θ*-burst durations, *d_δ_* and *d_θ_*. We rank burst durations in ascending order, with the shortest duration corresponding to the smallest rank, and examine the scatter plots between the ranks of consecutive *d_δ_* and *d_θ_* (Fig. 7a). We find that *δ*-bursts of high ranks (i.e. long durations) tend to be followed by *θ*-bursts of low ranks (i.e. short durations). This anti-correlated coupling appears to be a basic characteristic of dynamics as it is observed throughout the entire sleep-wake cycle in both dark and light periods [1].

To quantify the coupling between consecutive *δ*- and *θ*-burst durations we utilize Spearman’s correlation coefficient, which assesses monotonic relationships between two variables. The Spearman’s coefficient is positive when observations of two variables tend to have similar ranks, and negative if they tend to have opposite ranks. Our analyses show that the Spearman’s coefficient calculated for consecutive *δ*- and *θ*-burst durations is significantly negative (Fig. 7b), indicating anti-correlated coupling. This is verified by a surrogate test where the sequence of consecutive *δ*- and *θ*-burst durations is randomized [1].

## 3 Discussion

We studied the dynamical features of wake- and sleep-dominant brain rhythms across 48 h recordings of the sleep-wake cycle. We found that transient bursts in *θ* and *δ* cortical rhythms continuously occur during the sleep-wake cycle, and exhibit a complex temporal organization which is characterized by a remarkable duality of scale-invariant power-law distribution for *θ*-burst durations (active states) and Weibull distribution with a exponential characteristic time scale for *δ*-burst durations (quiet states), a behavior typical of non-equilibrium systems self-organizing at criticality.

Importantly, we showed that active and quiet states are anti-correlated, and demonstrated that this coupling is essential part of the mechanism responsible for the emergent critical dynamics. The presence of such coupling is also manifested through the scale-invariant structure in the quiet times separating consecutive active states (*θ*-bursts) above a given duration, which we find to be described by a unique scaling function (generalized Gamma distribution). This structure links, across time scales, the duration of a given *θ*-burst with the time of its occurrence. Moreover, we found that sequences of consecutive *θ*- or *δ*-burst durations are long-range power-law correlated, indicating a scale-invariant organization in the temporal order of burst durations and a unique underlying process with persistent ‘memory’ spanning over a wide range of scales that statistically couples the duration of a given burst with the durations of hundreds of following bursts.

Our empirical analyses showed that the reported characteristics of active and quiet state dynamics are independent of the scale of observation or on the threshold used to identify bursts, and remain continuously present during dark and light periods [1]. The presence of multiple scale-invariant characteristics related to distributions, correlations, coupling and timing of bursting events, is a strong evidence for criticality underlying cortical dynamics across sleep and wake.

Further, we demonstrated that the temporal structure characterizing the alternation of active and quiet states is closely reminiscent of the temporal organization of earthquakes. Indeed, we found that the distributions of quiet times between consecutive *θ*-bursts (active states) above a given duration threshold follow a unique scaling function, which is well described by a generalized Gamma distribution. This distribution is the universal scaling function for the distribution of waiting times between consecutive earthquakes, independently of geographical location and minimum detection thresholds [21, 25]. Moreover, the reported anticorrelated coupling between active and quiet states has been also found in earthquake dynamics [25].

All these features outline a general picture unifying previous empirical observations of spontaneous neuronal network dynamics at different levels, from networks of dissociated cortical neurons [30] and local field potentials (LFP) in cortex slice cultures [2] and awake monkeys[ 11], to the human brain [31–34], and the dynamics of sleep-stage and arousal transitions across species [7, 8, 35, 36] — where either distributions or temporal correlations of active events have been studied and discussed in the context of self-organized criticality. Crucially, our analyses show that both power-law distributions and long-range correlations emerge through specific temporal relation and coupling between active and quiet states, suggesting that they are distinctive characteristics of a class of systems self-organizing at criticality.
